# Supplementary material for: Topical Antibiotic Prophylaxis and Intravitreal Injections: Impact on the Incidence of Acute Endophthalmitis—A Nationwide Study in France from 2009 to 2018
Source: Pharmaceutics. 2022 Oct 7;14(10):2133. doi: 10.3390/pharmaceutics14102133 (PMC9611403; doi:10.3390/pharmaceutics14102133)
Supplement: Supplementary file 1 [file pharmaceutics-14-02133-s001.zip › pharmaceutics-1937922-supplementary.pdf]

pharmaceutics

MDPI

Supplementary Materials: Topical Antibiotic Prophylaxis and Intravitreal Injections: Impact on the Incidence of Acute Endophthalmitis—A Nationwide Study in France from 2009 to 2018

Florian Baudin, Eric Benzenine, Anne-Sophie Mariet, Inès Ben Ghezala, Alain M. Bron, Vincent Daien, Pierre-Henry Gabrielle, Catherine Quantin and Catherine Creuzot-Garcher

Variable	Univariate Poisson regression		Multivariate Poisson regression	
	IRR (95% CI)	*P*	*IRR (95% CI)*	*P*
**Age category (reference = under 70), years**		**<0.001**		
70–79	0.93 (0.80–1.08)			
80–84	0.78 (0.66–0.92)		**Age category (reference = under 84), years**	
85+	0.71 (0.60–0.83)		0.88 (0.77–1.02)	**0.07**
**Sex (reference = female)**				
Male	0.97 (0.87–1.09)	0.62	**0.87 (0.77–0.99)**	**0.03**
**Diabetes (reference = no diabetes)**				
Diabetes	1.07 (0.95–1.21)	0.26		
**Insulin-dependent (reference = non-insulin-dependent diabetes)**				
Insulin-dependent diabetes	1.10 (0.90–1.34)	0.35		
**Topical antibiotic prophylaxis (reference = none)**				
Topical antibiotic prophylaxis	1.11 (0.99–1.24)	0.06		
**Antibiotic prophylaxis (reference = none)[a]**				
Aminoglycosides	1.36 (1.18–1.57)	**<0.001**	Aminoglycosides associated with topical steroids: **1.50 (1.21–1.86)**	**0.002**
Macrolides	0.97 (0.87–1.09)	0.64		
Fluoroquinolones	1.22 (1.05–1.42)	**0.01**	1.16 (0.98–1.37)	0.11
Fusidic acid	1.32 (0.33–5.28)	0.70		
Polypeptides	0.83 (0.12–5.92)	0.85		
Rifamycins	1.58 (0.78–3.18)	0.30		
**Antibiotics associated with steroids (reference = none)**	**1.89 (1.57–2.27)**	**<0.001**		
**Agent class (reference = anti-vascular endothelial growth factor agents)**				
Corticosteroids	3.55 (2.99–4.22)	**<0.001**		
**Agent preparation (reference = non-prefilled ranibizumab)[b]**		**<0.001**		**<0.001**
Prefilled ranibizumab	**0.82 (0.70–0.97)**		**0.85 (0.72–0.99)**	
Aflibercept	1.09 (0.94–1.27)		1.13 (0.97–1.31)	
Pegaptanib	1.56 (0.50–4.88)		1.51 (0.48–4.70)	
Dexamethasone	**2.83 (2.30–3.50)**		**2.86 (2.31–3.53)**	
Triamcinolone	**10.83 (7.69–15.26)**		**10.23 (7.25–14.43)**	
**Year (reference = 2009)**		**<0.001**		
2010	1.13 (0.81–1.58)			
2011	0.86 (0.62–1.21)			
2012	0.68 (0.49–0.95)			
2013	0.77 (0.56–1.05)			
2014	0.65 (0.48–0.89)			
2015	0.64 (0.47–0.87)			
2016	0.63 (0.47–0.85)			
2017	0.62 (0.46–0.84)			
2018	0.83 (0.62–1.11)			

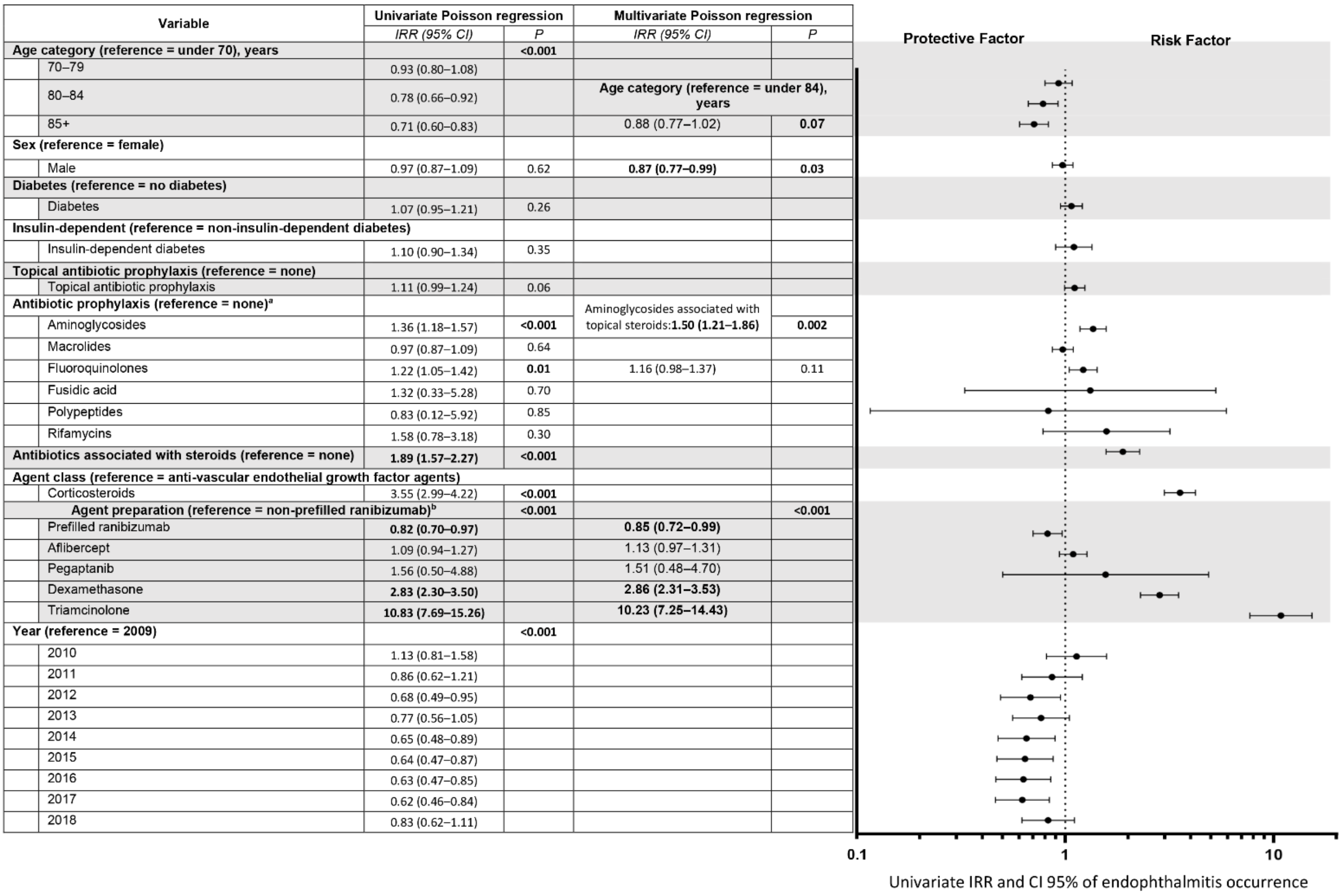

Figure S1. Study Design.
